# PRMT5 inhibition sensitizes glioblastoma tumor models to temozolomide

**DOI:** 10.21203/rs.3.rs-5936706/v1

**Published:** 2025-02-10

**Authors:** Shumpei Onishi, Sridharan Jayamohan, Ashis Chowdhury, Sarah Rivas, Yoshihiro Otani, Sara A. Murphy, Kimberly A. Rivera-Caraballo, Stuart Walbridge, Ashish H. Shah, Bayu Sisay, Dragan Maric, Abdel Elkahloun, Kory Johnson, John Heiss, Tae Jin Lee, Sangamesh G. Kumbar, Desmond A. Brown, Ji Young Yoo, Andrew Brenner, Balveen Kaur, Gangadhara R. Sareddy, Yeshavanth Kumar Banasavadi-Siddegowda

**Affiliations:** NINDS, NIH; University of Texas Health San Antonio; NINDS, NIH; NINDS, NIH; University of Texas Health Science Center at Houston; Augusta University Medical Center; Augusta University Medical Center; NINDS, NIH; NINDS, NIH; National Human Genome Research Institute, National Institutes of Health; NINDS, NIH; National Human Genome Research Institute, National Institutes of Health; NINDS, NIH; NINDS, NIH; University of Texas Health Science Center at Houston; University of Nebraska Medical Center; NINDS, NIH; University of Texas Health Science Center at Houston; Mays Cancer Center at University of Texas Health San Antonio; Augusta University Medical Center; University of Texas Health San Antonio; NINDS, NIH

**Keywords:** PRMT5, TMZ, LLY283, DNA damage repair, Glioblastoma

## Abstract

**Background:**

Despite multi-model therapy of maximal surgical resection, radiation, chemotherapy, and tumor-treating fields, glioblastoma patients show dismal prognosis. Protein Arginine Methyltransferase 5 (PRMT5) is overexpressed in glioblastoma and its inhibition imparts an anti-tumor effect. Even though Temozolomide (TMZ) is the standard chemotherapeutic agent in the treatment of glioblastoma, tumor cells invariably develop resistance to TMZ. However, the mechanistic role of PRMT5 in glioblastoma therapy resistance is unknown.

**Methods:**

Patient-derived primary glioblastoma neurospheres (GBMNS), treated with PRMT5 inhibitor (LLY-283) or transfected with PRMT5 target-specific siRNA were treated with TMZ and subjected to *in vitro* functional and mechanistic studies. The intracranial mouse xenograft model was used to test the *in vivo* antitumor efficacy of combination treatment.

**Results:**

We found that PRMT5 inhibition increased the cytotoxic effect and caspase 3/7 activity of TMZ in GBMNS suggesting that apoptosis is the potential mode of cell death in the combination treatment. PRMT5 inhibition abrogated the TMZ-induced G2/M cell cycle arrest. Unbiased transcriptomic studies indicate that PRMT5 inhibition negatively enriches DNA damage repair genes. Importantly, combination therapy increased DNA double-strand breaks (H2AX foci) and enhanced the DNA damage (comet assay), suggesting that the combination treatment increases the TMZ-induced DNA damage. Specifically, the LLY-283 treatment blocked homologous recombination repair in GBMNS. *In vivo*, LLY-283 and TMZ combination significantly curbed the tumor growth and prolonged the survival of tumor-bearing mice.

**Conclusion:**

Concomitant treatment of LLY-283 and TMZ has significantly greater antitumor efficacy, suggesting that PRMT5 inhibition and TMZ combination could be a new therapeutic strategy for glioblastoma.

## Statement of Significance

This is the first study to test the effect of concomitant inhibition of PRMT5 and TMZ treatment in glioblastoma models. PRMT5 inhibition attenuates the DNA damage repair leading to sensitization of GBMNS to TMZ. As TMZ is the first-line chemotherapeutic agent in treating glioblastoma, this study has significant clinical application to overcome TMZ resistance as LLY-283 is a potent brain penetrant PRMT5 inhibitor.

## Introduction

Glioblastoma is the most common malignant primary brain tumor and is classified under CNS WHO grade 4 neoplasms. The standard therapeutic approach to glioblastoma involves aggressive multi-model therapy including maximum safe surgical resection followed by radiation and chemotherapy. The prognosis is dismal, and the median survival is around 15–20 months because of tumor progression and recurrence (1, 2). Apart from intra-tumoral heterogeneity, acquired therapy resistance has been implicated in treatment failure (3–5). A key factor contributing to therapy resistance is the intrinsic DNA repair capacity of tumor cells, particularly glioma stem-like cells, also known as glioma-initiating cells (6, 7). These cells exhibit dysregulated DNA damage response pathways (8–10).

While the Type II arginine methyltransferase, Protein arginine methyltransferase 5 (PRMT5), through symmetric di-methylation of histone and non-histone protein arginine proteins, regulates numerous cellular functions, its expression is dysregulated in glioblastoma (11). In our previous study, we and others have shown that the PRMT5 that is overexpressed in glioblastoma inversely correlates with patient survival (11, 12), and inhibition of PRMT5 caused apoptosis in differentiated glioblastoma tumor cells and senescence in stem-like glioblastoma tumor cells (13). As PRMT5 is a druggable target, several PRMT5 inhibitors including LLY-283 are under investigation for treating glioblastoma (14, 15). Recent studies showed that PRMT5 regulates homologous recombination (HR) repair through methylation of RUVBL1 and histone arginine residues (16, 17). Further, PRMT5 contributes towards DNA repair by regulating the histone-modifying enzymes through alternative splicing (18), activation of epigenetic activators (19) and promotion of non-homologous end joining (NHEJ) by stabilizing and methylating 53BP1 (20).

Temozolomide (TMZ) is a DNA alkylating agent that induces the alkylation of guanine at the O6 position which results in DNA damage and cytotoxic effect (21). The anti-tumor efficacy of TMZ for glioblastoma was validated in 2005 for newly diagnosed glioblastoma cases. Since then, it has been utilized as the first-line chemotherapeutic intervention for glioblastoma (1). Despite its anti-glioblastoma effect, TMZ resistance and subsequent recurrence/progression of the tumor is inevitable (22). Traditionally, TMZ-resistance was primarily linked to the MGMT status of the tumor cells. But recent studies have identified alternative factors that significantly contribute to TMZ-resistance: (i) Intrinsic ability of glioma stem-like cells to repair TMZ-induced DNA damage (6, 23, 24), (ii) epigenetic modifications and (iii) signaling cascade dysregulation (25) are some of the major contributors towards TMZ resistance mechanism thus increasing the complexity of TMZ-resistance.

With the backdrop of the enhanced intrinsic ability of glioma stem-like cells to repair the damaged DNA and the contribution of DNA damage repair mechanisms for TMZ-resistance, we tested if the inhibition of PRMT5 alters the TMZ-resistance in glioma stem-like cells via attenuation of DNA damage repair. In this study, we show that PRMT5 inhibition suppresses HR repair of glioblastoma, leading to increased TMZ-induced DNA damage, and enhances the antitumor efficacy in both *in vitro* and *in vivo* glioblastoma tumor models.

## Materials and Methods

### Cell Culture:

The patient-derived primaryglioblastoma neurospheres (GBMNS) GSC040815 and GSC082209 were developed as described previously (26), and GBM12 and GBM43 were obtained from Dr. Jann Sarkaria’s laboratory (Mayo Clinic, Rochester, MN). The cells were cultured as neurospheres in DMEM/F12 medium without phenol red (Invitrogen, Carlsbad, CA, USA), supplemented with 1% penicillin-streptomycin, 50 ng/mL fibroblast growth factor (FGF), 50 ng/mL epidermal growth factor (EGF), 2% B-27 supplement without vitamin A (Invitrogen), and 1% sodium pyruvate (Fisher Scientific, Hampton, NH, USA). Cultures were maintained in low-attachment flasks. Cells were dissociated using TrypLE Express (Invitrogen), authenticated via short tandem repeat profiling, and screened for mycoplasma contamination, with all cultures testing negative.

### LLY-283:

LLY-283 was purchased from Selleck Chemicals LLC (Houston, TX) and was reconstituted as per the manufacturer’s recommendation.

### Cell-titer glo Assay:

The CellTiter-Glo Luminescent Cell Viability Assay (Promega, Madison, WI) was performed following the manufacturer’s protocol to assess cell viability and/or proliferation. Luminescence was measured using a Biotek FLx800 microplate reader.

### PRMT5 siRNA Transfection:

GBMNS were transfected with either control siRNA (Non-Target Scrambled, Cntrl) or PRMT5-target-specific siRNA (P5i) (Dharmacon, Lafayette, CO, USA) using RNAiMAX Lipofectamine and Opti-MEM (Invitrogen, Carlsbad, CA, USA) according to the manufacturer’s instructions.

### Cell cycle analysis:

Cells were treated with respective treatment conditions. GSC040815 and GSC082209 were treated with 50 μM and GBM43 and GBM12 were treated with 6 μM of TMZ. After 48 hours, the cells were washed with phosphate-buffered saline (PBS) and fixed with 80% ethanol. Subsequently, the fixed cells were stained with 50 μg/mL propidium iodide (PI) (Sigma-Aldrich). Flow cytometric analysis was performed using a Becton Dickinson LSRII fluorescence-activated cell sorter (FACS) (Becton-Dickinson, San Jose, CA) and/or a MoFlo Astrios EQ cell sorter (Beckman Coulter, GA). Data were analyzed using Modfit software (Topsham, ME).

### Western Blot:

Cells were lysed with RIPA buffer (Sigma, St. Louis, MO, USA) containing a protease/phosphatase inhibitor cocktail at 1X concentration (Cell Signaling, Danvers, MA, USA). Protein concentration was determined using the BioRad Protein Assay Kit (Bio-Rad, Hercules, CA, USA). Equal amounts of protein were denatured with 1X NuPAGE reducing agent and 1X NuPAGE LDS sample buffer, then loaded onto 4–12% Tris-Bis gels. Proteins were transferred to nitrocellulose membranes (Invitrogen, Carlsbad, CA, USA). Antibodies against PRMT5 and Tubulin were obtained from Abcam (Cambridge, UK), while antibodies for H4R3, PCNA, GAPDH, APEX1, RAD23B, RAD51, and POLD1 were purchased from Cell Signaling (Danvers, MA, USA). All antibodies were used at a 1:1000 dilution.

### Caspase 3/7 Activity Assay:

GBMNS were seeded into 96-well plates and treated with LLY-283, TMZ, or the combination of LLY-283 + TMZ. GSC040815 and GSC082209 were treated with 50 μM of TMZ and 50 μM of LLY-283. GBM43 and GBM12 were treated with 6 μM of TMZ and 3 μM of LLY-283. 48 hours post-treatment, caspase 3/7 activity was assessed using the Caspase-Glo^®^ 3/7 Assay System (Promega, Madison, WI, USA) following the manufacturer’s instructions. For PRMT5-intact and depleted cells, 48 hours post-transfection, GBMNS were seeded in 96 well plates and treated with increasing doses of TMZ. 48 hours post-treatment caspase 3/7 activity was measured.

### RNA-sequencing:

GBMNS were treated with LLY2–83 (50 μM), TMZ (50 μM), or a combination of LLY-283 and TMZ. Twenty-four hours post-treatment, cells were collected, and RNA was extracted using the RNeasy Mini Kit (Qiagen). RNA sequencing (RNA-seq) experiments were conducted as previously described (26), utilizing the NextSeq 1000/2000 P2 system. The biological significance of these genes was analyzed through Gene Ontology (GO) and Gene Set Enrichment Analysis (GSEA) (27, 28). RNA-seq results have been deposited in the GEO database (https://www.ncbi.nlm.nih.gov/geo/query/acc.cgi?acc=GSE286560)

### qPCR-based HR Assay:

The HR Assay Kit (Norgen Biotek, Ontario, Canada) was used to assess HR efficiency following the manufacturer’s instructions and as described previously.(26) GBMNS transfected with the HR kit plasmids using Lipofectamine 3000 (Thermo Fisher Scientific). Six hours post-transfection, the GBMNS were treated with LLY-283 (50 μM), TMZ (50 μM), or the combination for 48 hours. Genomic DNA was then extracted using the DNA Purification Kit (Qiagen, Germantown, MD) according to the manufacturer’s protocol. Quantitative PCR (qPCR) was performed using the QuantStudio 6 Flex system (Life Technologies).

### H2AX Foci Assay:

GBMNS were seeded onto Geltrex-coated (Thermo Fisher Scientific, Waltham, MA) Lab-Tek II chamber slides (Thermo Fisher Scientific, Waltham, MA) and incubated overnight. The cells were treated with either a control (DMSO), LLY-283, TMZ, or a combination of LLY-283 and TMZ. GSC040815 and GSC082209 were treated with 50 μM of TMZ and 50 μM of LLY-283. GBM43 and GBM12 were treated with 6 μM of TMZ and 3 μM of LLY-283. PRMT5-intact and depleted GBMNS were seeded onto Geltrex-coated chamber slides and treated with DMSO or TMZ. GSC040815 and GSC082209 were treated with 50 μM and GBM43 and GBM12 were treated with 6 μM of TMZ. 48 hours post-treatment, the samples were fixed with 4% paraformaldehyde (Electron Microscopy Sciences, Hatfield, PA) for 20 minutes and permeabilized with 0.1% Triton X-100 for 10 minutes. Immunofluorescence blocking buffer (Cell Signaling Technology, Danvers, MA) was used to block the cells for 1 hour at room temperature. The cells were then incubated overnight at 4 °C with γH2AX antibody (Cell Signaling Technology, Danvers, MA) at 1:500 dilution. Slides were then incubated with Alexa Fluor 594-conjugated secondary antibody (Abcam, Waltham, MA) (1:200 dilution) for 1 hour at room temperature. Coverslips were mounted onto the slides using a vectashield antifade mounting solution with DAPI (Vector Laboratories, Newark, CA). Images of the γH2AX foci were captured using a confocal microscope (Leica Microsystems, Morrisville, NC), and the foci were counted manually.

### Single Cell Alkaline Gel Electrophoresis (Comet Assay):

PRMT5-depleted or LLY-283-treated GBMNS were treated with either vehicle (0.1% DMSO v/v), or TMZ or the combination (P5i + TMZ, LLY-283 + TMZ). For the LLY-283/TMZ treatment condition, GSC040815 and GSC082209 were treated with 50 μM of TMZ and 50 μM of LLY-283. GBM43 and GBM12 were treated with 6 μM of TMZ and 3 μM of LLY-283. GSC040815 and GSC082209 were treated with 50 μM and GBM43 and GBM12 were treated with 6 μM of TMZ for PRMT5 knockdown experiment. Forty-eight hours post-treatment, single-cell suspensions of GBMNS were seeded in low-melting agarose (20×10^^5^ cells/mL) and 50 μl of the cell suspension was dispersed on the pre-treated microscope slides. Alkaline lysis (1 hour) and DNA unwinding (20 minutes) were performed before electrophoresis. Electrophoresis was done at 21V constant for 40 min in cold alkaline buffer. Samples were then dehydrated in 70% ethanol and air-dried for 15 minutes in the dark at 37°C incubator. Subsequently, the slides were stained with SYBR Gold (R & D Systems, Minneapolis, MN) for 30 minutes. Comet images were captured using the EVOS fluorescence microscope. At least 20 representative comets were measured for each treatment group.

### Schematic Diagrams:

The Schematic diagrams (Fig. 3H, Fig. 6A, and Fig. 6D) were generated using the BioRender software program Scientific Image and Illustration Software | BioRender (Toronto, Ontario, Canada).

### Intracranial injections:

Ethics Statement:The animal study was conducted following UT Health San Antonio IACUC approval and guidelines. Animal studies: NOD.CB17*-Prkdc*^*scid*^/NCrCrl mice, aged 6–8 weeks were purchased from Charles River (Wilmington, MO). GFP-Luciferase expressing GSC040815 (GSC040815 GFP-Luc) (1X10^4^ cells/mice) were implanted in the mice intracranially. Day 4, post-tumor implantation, mice were randomized to receive either vehicle (0.5% methylcellulose, 0.5% Tween 80 or 1:1 OraPlus: OraSweet), LLY-283 (50 mg/kg body weight/day in 0.5% methylcellulose, 0.5% Tween 80), TMZ (10 mg/kg body weight in 1:1 OraPlus: OraSweet ), or in combination via oral gavage. 50 mg/kg of LLY-283 was administered orally in weekly cycles of 3 days on, and 4 days off, until all the mice in the TMZ-treatment alone group reached the end stage of the study. Mice were treated with TMZ on days 7, 9, 11, 13, and 15 post-tumor implantation by oral gavage. The Xenogen IVIS system was used to follow the tumor growth. The mice were monitored regularly for neurological symptoms from the time of tumor implantation till they reached the experimental endpoint. Once they reached the end stage of the study, mice were euthanized and recorded their survival.

### Statistical analysis:

Statistical analyses were performed using GraphPad Prism software. A two-sided unpaired Student’s t-test was employed to determine statistical significance between two continuous groups, with results presented as mean values ± standard deviation. Survival curves were plotted using the Kaplan-Meier method, and statistical significance was assessed with the log-rank test. The Benjamini-Hochberg procedure was applied to adjust for multiple comparisons in post-hoc analyses. To further assess the survival benefit of the LLY283 + TMZ combination, a Cox proportional hazards regression model was fitted, and hazard ratios were calculated as described previously (29). A p-value of <0.05 was considered indicative of statistical significance.

## Results

### PRMT5 inhibition increases the sensitivity of TMZ in GBMNS

**Figure 1:**
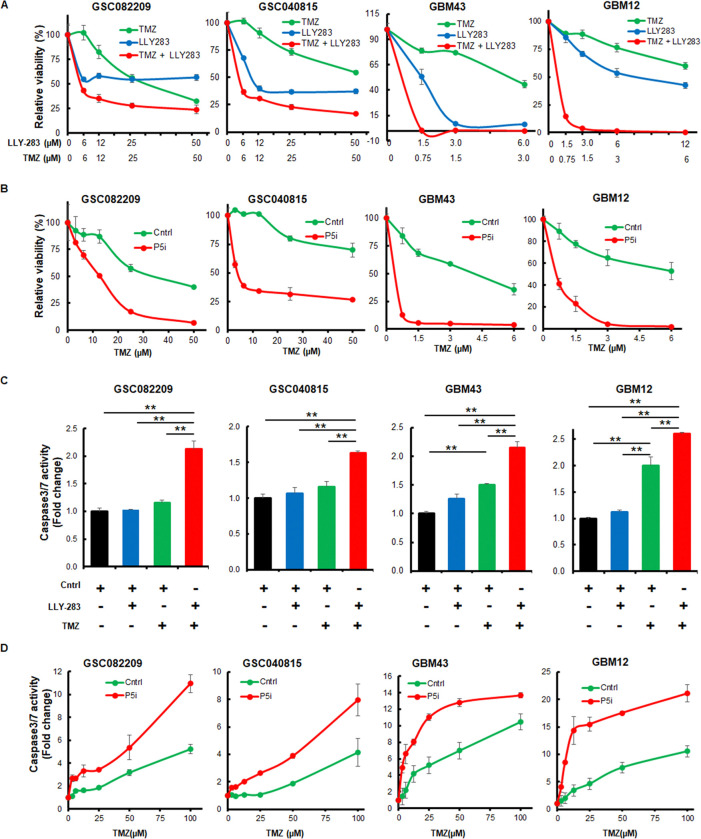
PRMT5 inhibition increases the sensitivity of TMZ in GBMNS: (A) Indicated GBMNS were treated with either TMZ and/or LLY283. 4 days post-treatment viability of the cells was measured by cell-titer glo assay. (B) GBMNS transfected with scrambled (Cntrl), or PRMT5-target specific siRNA (P5i) were treated with increasing doses of TMZ. 4 days post-treatment, cells were subject to viability assay (C) GBMNS were treated with TMZ and/or LLY283 for 48 hours and caspase3/7 activity was measured. (D) PRMT5-intact and depleted GBMNS were treated with increasing doses of TMZ, and caspase3/7 activity was measured 48 hours post-treatment with TMZ. n=3 (** *p*≤0.001).

To test if PRMT5 alters the effect of TMZ in glioblastoma, we used GBMNS that are relatively TMZ-resistant (GSC040815 and GSC082209) and TMZ-sensitive (GBM12 and GBM43). Initially, to confirm the inhibition of PRMT5 activity by LLY-283 we probed for the expression of H4R3 (Suppl. Fig. 1A). We treated GBMNS with increasing doses of TMZ and/or LLY283 (Fig. 1A). While the LLY283 treatment reduced the EC50 value of TMZ from more than 30 μM to less than 5 μM in GSC 082209 and 040815, the EC50 value of TMZ is reduced from 6 μM to less than 1 μM in the TMZ-sensitive GBM12 and GBM43. To further validate this finding, we depleted PRMT5 in the GBMNS using PRMT5-target specific siRNA (P5i) and treated them with increasing doses of TMZ (Suppl. Fig. 1B & Fig 1B). With PRMT5-depletion, the effective concentration of TMZ required to bring down the viability by 50% was reduced by at least 25-fold.

As the combination treatment of LLY-283 and TMZ decreased the viability of tumor cells significantly (Fig. 1A), we tested if it was because of apoptosis. GBMNS treated with LLY-283 and TMZ were subject to caspase 3/7 activity assay (Fig. 1C). With the lower doses of LLY283 or TMZ treatment alone, we found a minimal increase in the caspase 3/7 activity. But with the combination of lower doses of LLY283 and TMZ, caspase 3/7 activity spiked significantly, by 2-fold compared to control. Further, PRMT5-intact, and depleted cells were treated with TMZ; 48 hours post-treatment they were probed for caspase 3/7 activity (Fig. 1D). As expected, with TMZ treatment alone there was a dose-dependent increase in the caspase 3/7 activity, and it significantly increased with PRMT5-depletion. These results together confirm that PRMT5 inhibition sensitizes GBMNS to TMZ and enhances the TMZ-induced cytotoxic effect.

### PRMT5 inhibition abrogates the TMZ-induced G2/M cell cycle arrest.

**Figure 2:**
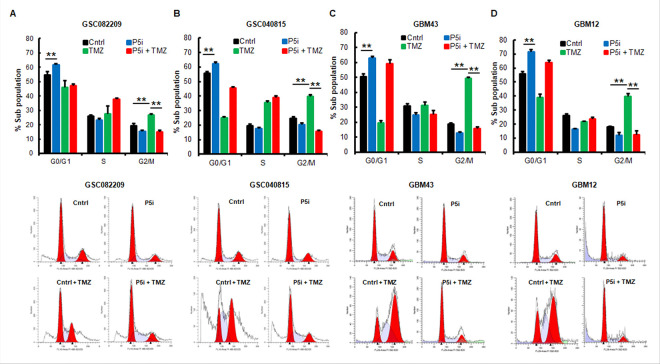
PRMT5 inhibition abrogates TMZ-induced G2/M cell cycle arrest: (A-D) PRMT5-intact and depleted GBMNS treated with TMZ for 48 hours were analyzed for the cell cycle progression using PI. (Upper panels) The graph represents the percent of the cell population in each stage of the cell cycle (**p ≤ 0.001). (Lower panels) Representative cell cycle histogram for each treatment condition across all the cell types that were analyzed for cell cycle progression.

Our earlier studies show that PRMT5 inhibition causes G1 cell cycle arrest in GBMNS.(13, 14) TMZ causes DNA damage, and the subsequent G2/M cell cycle arrest is the response by the affected cells to correct the damaged DNA (30, 31). Additionally, GBMNS also known as glioma initiating cells or glioma stem-like cells have high DNA repair capacity and are a significant contributing factor for TMZ therapy resistance (6, 7). Hence, we tested if PRMT5 inhibition affects the TMZ-induced G2/M cell cycle arrest. PRMT5-intact and depleted GBMNS were treated with TMZ (Fig. 2). 48 hours post-treatment, we subjected the cells to cell cycle analysis. As expected, PRMT5 inhibition caused G1 cell cycle arrest and TMZ induced the G2/M cell cycle arrest. Interestingly, PRMT5-depletion nullified the G2/M cell cycle arrest induced by TMZ, suggesting that PRMT5 might play a role in helping the repair of the DNA damaged by TMZ.

### PRMT5 regulates DNA damage repair in GBMNS.

**Figure 3:**
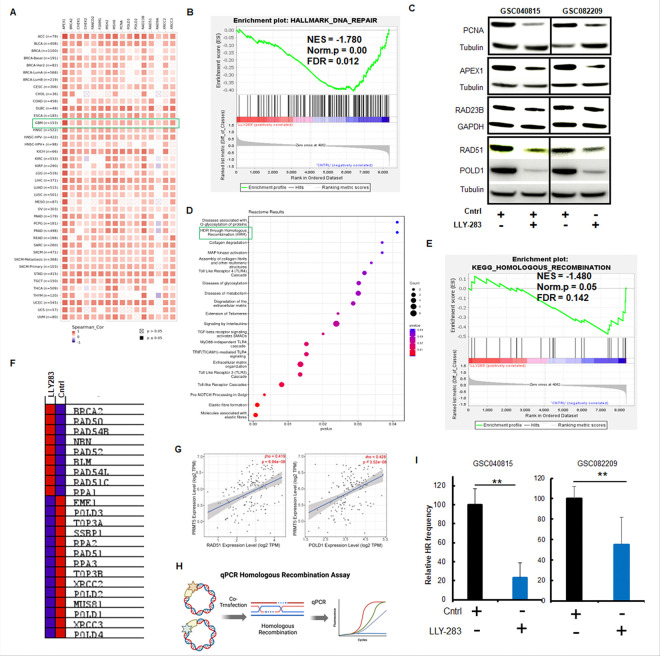
PRMT5 inhibition downregulated DNA damage repair genes in GBMNS: (A) Heatmap showing the correlation between PRMT5 and DNA repair genes across multiple tumor types. (B) GSC082209 treated with Control (DMSO) or LLY-283 for 24 hours were subjected to RNA sequencing. GSEA enrichment plot shows the correlation of LLY-283 with pan-DNA damage repair genes. (C) GSC040815 and GSC082209 treated with LLY-283 (50 μM) were probed for indicated DNA damage repair proteins by western blot. (D) Top differentially expressed genes based on the RNA sequencing analysis of panel B. (E) GSEA enrichment analysis showing a negative correlation between LLY-283 treatment and HR repair gene set. (F) Heatmap showing the genes that are differentially expressed based on panel E gene enrichment analysis. (G) Scatter plot from TIMER2.0 database showing correlation between PRMT5, and HR genes (RAD51 and POLD1) based on glioblastoma TCGA data sets. p-value computed for each data set. (H) Schematic representation of qPCR-based HR assay protocol. (I) GSC040815 and GSC082209 treated with LLY-283 were subjected to HR activity. n=3 (** *p*≤0.001).

As we observed the abrogation of TMZ-induced G2/M cell cycle arrest with PRMT5 inhibition, we hypothesized that PRMT5-inhibition-induced sensitization of TMZ is because of the suppression of DNA repair genes by PRMT5. Moreover, there is abundant literature linking PRMT5 to DNA damage repair machinery (16–20). To investigate the association between PRMT5 and DNA damage repair genes across various types of cancer, we analyzed the correlation between the expression levels of PRMT5 and DNA damage repair genes using TIMER2.0 with data from The Cancer Genome Atlas (TCGA). Our analysis revealed that PRMT5 expression is positively correlated with DNA damage repair genes across multiple tumor types, including glioblastoma (Fig. 3A).

To gain mechanistic insights into the combination effect, we conducted RNA-sequencing analysis on the GBMNS treated with LLY-283 or TMZ or the combination of TMZ and LLY-283 to screen the global transcriptional changes. Initially, we compared the gene expression profile of the LLY-283 treatment. GSEA analysis showed negative enrichment of genes involved in DNA repair pathways with LLY-283 treatment (Fig. 3B and Suppl. Fig. 2). To confirm this result, we probed for some of the DNA repair genes by western blot (Fig. 3C). LLY283 treatment reduced the expression of DNA repair genes such as PCNA, RAD51, POLD1, APEX1 and RAD23B, thus validating the RNA sequencing data.

One of the top downregulated pathways with LLY-283 is Homology-directed Repair (HDR) through HR (Fig. 3D). Incidentally, GSEA analysis showed negative enrichment for the HR genes (Fig. 3E and 3F). To understand the clinical relevance of HR genes, we analyzed the TCGA patient database and found a positive correlation between PRMT5 expression and HR genes such as RAD51 and POLD1 (Fig. 3G) that were downregulated with LLY-283 treatment (Fig. 3B and 3C). To further reconfirm the HR repair in the context of LLY-283 treatment in GBMNS, we conducted the HR repair assay (Fig. 3H and 3I). LLY283 treatment resulted in a significant decrease in HR repair of GBMNS. HR assay (Fig. 3H and 3I). These results together suggest that LLY-283 treatment negatively affects the DNA repair gene sets in general and HR in particular.

### PRMT5 inhibition enhances the TMZ-induced DNA damage.

**Figure 4:**
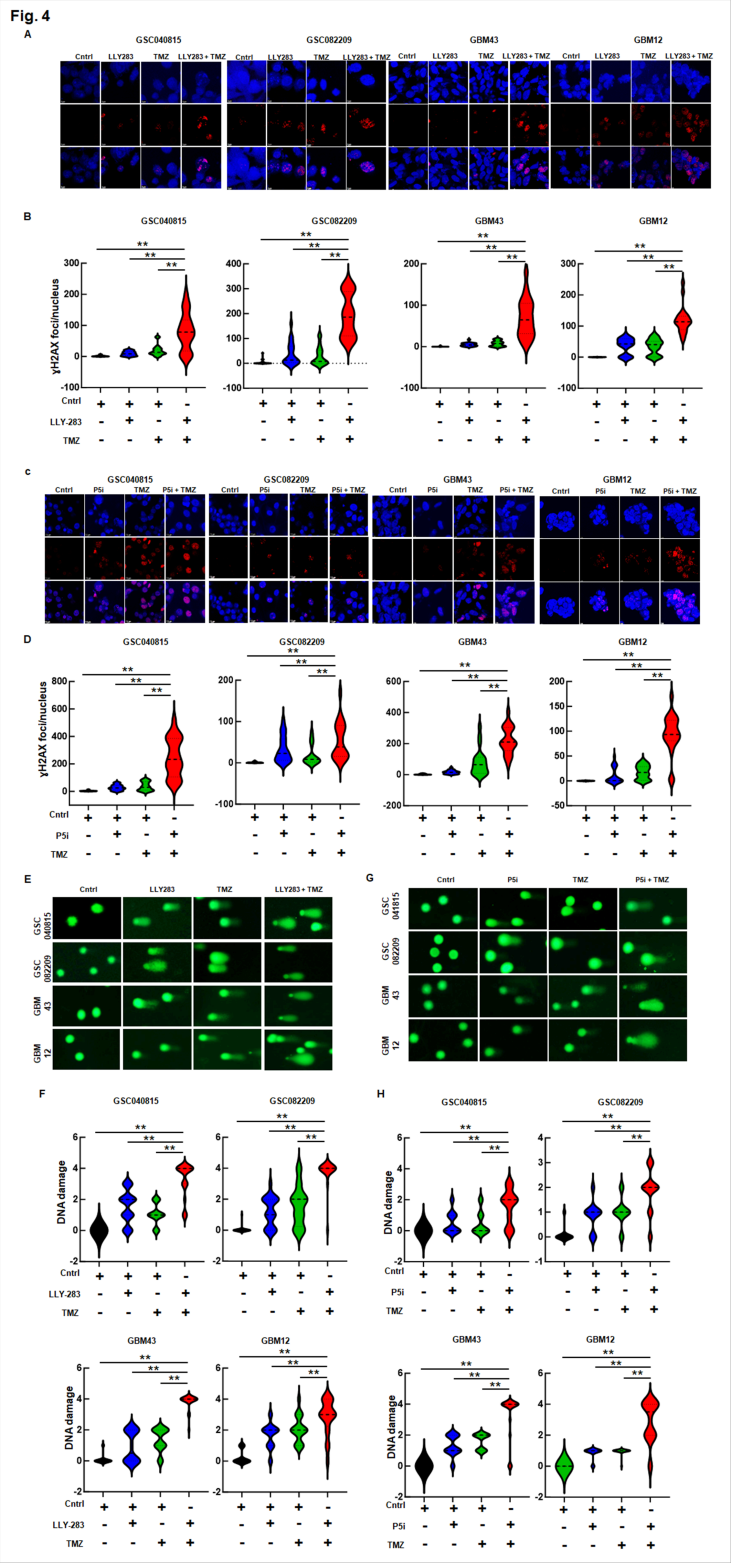
PRMT5 inhibition enhances the TMZ-induced DNA damage: (A, B) GBMNS treated with LLY-283 and/or TMZ for 48 hours were probed for H2AX foci by immunofluorescence and quantified. (**p ≤ 0.001). (C, D) PRMT5 transfected cells (P5i) treated with TMZ were probed for H2AX foci by immunofluorescence and the number of foci were quantified manually. (**p ≤ 0.001). (E, F) GBMNS treated with LLY-283 and/or TMZ for 48 hours were subjected to comet assay. DNA damage was graded/quantified from 0 to 4 based on the tail length and comet head size. (0 = no, 1= mild, 2 = moderate, 3 = high, 4= very high DNA damage. (**p ≤ 0.001). (G, H) GBMNS transfected with PRMT5 (P5i) were treated with TMZ for 48 hours and were subjected to comet assay. DNA damage was graded/quantified from 0 to 4 based on the tail length and comet head size. (0 = no, 1= mild, 2 = moderate, 3 = high, 4= very high DNA damage. (**p ≤ 0.001).

As the database analysis and RNA-seq results implicated the role of PRMT5 in DNA damage repair, to test its role in the context of TMZ treatment, we treated GBMNS with LLY-283 and TMZ and probed for H2AX staining, a classical marker of DNA double-strand breaks (Fig. 4A and 4B). Sublethal doses of TMZ and LLY-283 increased the number of H2AX foci compared to control. With the combination treatment, there was a significant increase in the number of foci suggesting enhanced DNA damage. We also treated PRMT5-depleted GBMNS with TMZ and probed for H2AX foci. PRMT5 knockdown in combination with TMZ enhanced the H2AX foci formation (Fig. 4C and 4D).

To further confirm that PRMT5 inhibitor-mediated downregulation of DNA repair enhances TMZ-mediated DNA damage, we conducted the comet assay. GBMNS treated with LLY-283 and TMZ were subjected to comet assay (Fig. 4E and F). Semi-quantitative analysis of DNA damage in the form of tail length and size of the comet head showed that with LLY-283 or TMZ treatment alone, there was a significant increase in DNA damage. With the combination of LLY-283 and TMZ, the DNA damage was robust. Also, there was enhanced DNA damage with the treatment of PRMT5-depleted GBMNS with TMZ across all GBMNS tested (Fig. 4G and 4H). Together, these results confirm that PRMT5 inhibition potentiates TMZ-induced DNA damage.

### LLY-283 blocks the TMZ-induced HR repair in GBMNS.

**Figure 5:**
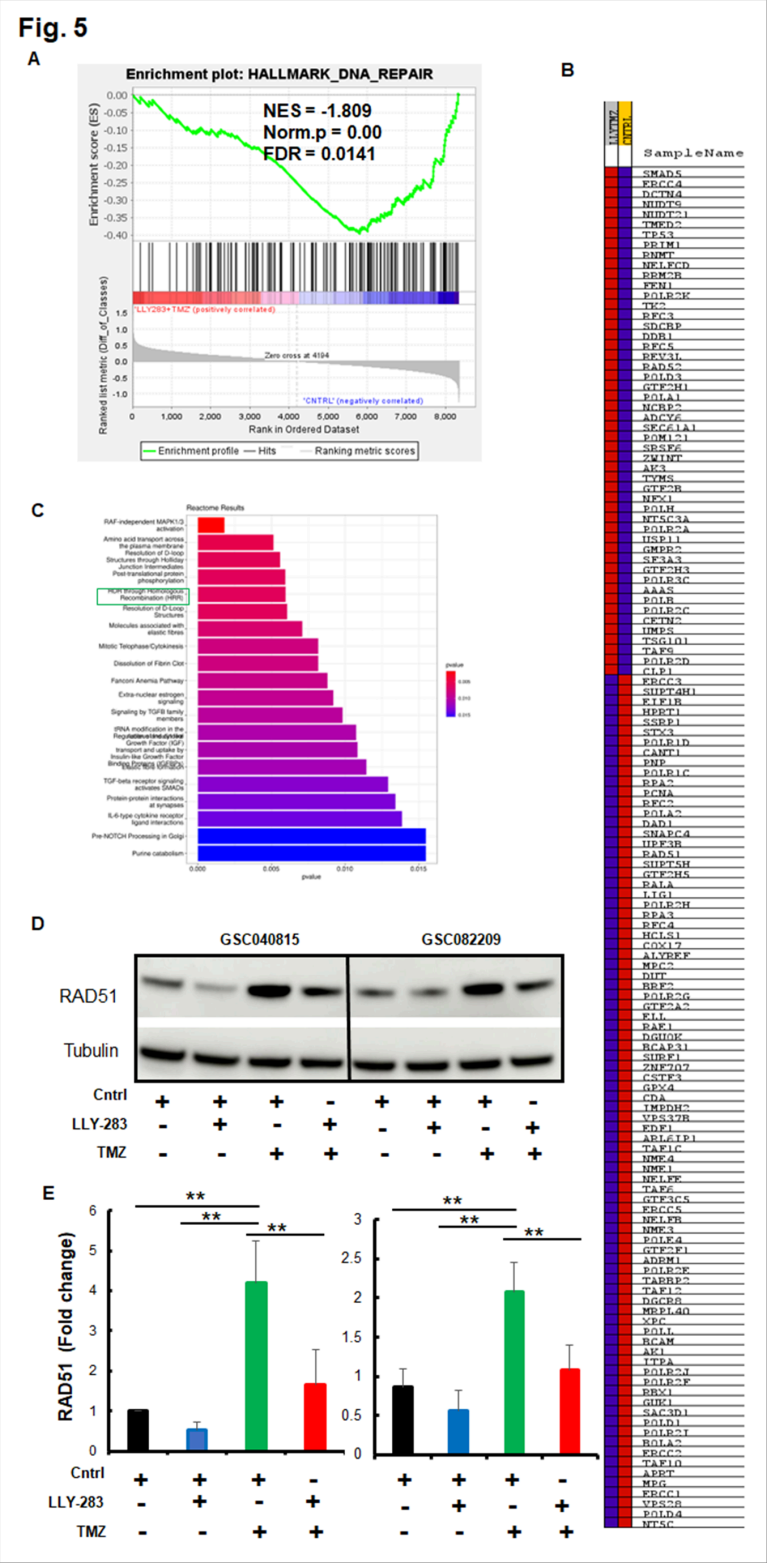
LLY-283 blocks the TMZ-induced HR repair in GBMNS: (A) GSEA enrichment analysis showing negative enrichment of DNA repair gene sets in GSC082209 treated LLY-283 and TMZ combination. (B) Heatmap for showing differential gene expression based on panel A. (C) Top gene ontology terms of differentially expressed genes pointing out HDR through HR. (D) GSC040815 and GSC082209 treated with LLY283 (50 μM), TMZ (50 μM), or the combination of LLY-283 and TMZ, were probed for HR marker RAD51 by western blot. (E) Quantification of panel D showing the expression of RAD51 (**p ≤ 0.001).

Having confirmed enhanced DNA damage with the combination therapy, we conducted gene enrichment analysis for the combination treatment condition (LLY283 + TMZ) (Fig. 5A). Combination treatment showed negative enrichments of DNA damage repair genes. Further, Reactome (Fig. 5B and 5C), WikiPathways and KEGG plot analysis (Suppl. Fig. 3) showed HR as one of the topmost pathways that were downregulated with the combination of LLY-283 and TMZ. To confirm this result, we probed for the HR marker RAD51 by western blot analysis (Fig 5D and 5E). While TMZ induced the expression of RAD51, LLY-283 downregulated it. These results suggest that LLY-283 blocks the TMZ-induced HR repair mechanism thus enhancing the TMZ-induced DNA damage and subsequent sensitization of GBMNS to TMZ.

### *In vivo*, PRMT5 inhibition enhances the antitumor efficacy of TMZ.

**Figure 6:**
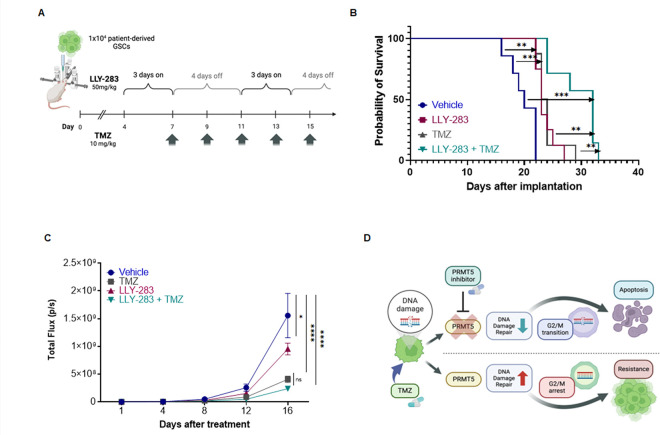
Combination of LLY-283 and TMZ enhances the in vivo antitumor efficacy: (A) Schematic representation of the *in vivo* study. (B) Mice were implanted with GSC040815 that expresses GFP-Luciferase. Post-implantation mice were treated with different treatment conditions and the Kaplan–Meier survival curve was plotted at the end of the study. (C) Quantification of the tumour volume based on the luciferase images generated during the study (D) Shown is the working model for the PRMT5-inhibition triggered apoptosis in the TMZ-treated GBMNS.

To assess the effect of combination therapy on tumor growth and survival, we used an intracranial GBM mouse model. GSC040815-Luc were implanted in the mice and were treated with LLY-283 and/or TMZ as detailed in Fig. 6A and materials and methods. Monotherapy with LLY-283 or TMZ increased the median survival of tumor-bearing mice from 20 days to 23 days (Fig. 6B). But the median survival of mice in the combination treatment increased to 33 days suggesting that the combination treatment has a better anti-tumor effect compared to LLY-283 or TMZ treatment alone. Further, we also followed the tumor growth (Fig. 6C). Treatment with LLY-283 and TMZ combination reduced the tumor growth significantly. These results together suggest that inhibition of PRMT5 enhances the antitumor efficacy of TMZ in GBMNS *in vivo*. The schematic representation (Fig. 6D) depicts the potential mechanism through which PRMT5 inhibition sensitizes GBMNS for TMZ.

## Discussion

The therapeutic outcome for glioblastoma is grave even with multimodal standard therapy that includes surgical resection followed by radiation and concurrent chemotherapy. Apart from tumor heterogeneity and activation of tumor escape pathways, the intrinsic ability of glioblastoma tumor cells to repair the damaged DNA induced by treatment plays a significant role in imparting radio- and/or chemotherapy resistance. The key to overcoming this issue is to explore the potential resistance mechanism and develop a therapeutic regime that combines drugs that synergize with each other to produce additional anti-tumor efficacy at lower, less toxic doses.

In this study we show that i) PRMT5 inhibition sensitizes the GBMNS to TMZ, ii) Inhibition of PRMT5 abrogates the TMZ-induced G2/M cell cycle arrest, iii) LLY-283 treatment downregulates the DNA-DSB repair pathway, particularly HR, iv) Inhibition of PRMT5 increases TMZ-induced DNA damage by blocking the DNA damage repair pathways, v) In vivo, the combination of LLY-283 and TMZ has more enhances the antitumor efficacy and prolongs the survival of tumor-bearing mice.

PRMT5 acts as a critical regulator of DNA damage repair through multiple molecular pathways to stabilize the genomic DNA and facilitate the DNA repair processes. PRMT5 through methylation of RUVBL1 plays an important role in coordinating double-strand break by HR (16). Further, evidence shows that PRMT5 mediates HR repair through histone arginine-methylation to maintain genomic stability (17). PRMT5, in coordination with pICln acts as an epigenetic activator of DNA double-strand break repair genes (19). Through the regulation of alternative splicing of histone-modifying enzymes, PRMT5 controls DNA repair (18). With the infliction of DNA damage, PRMT5 promotes NHEJ DNA repair through methylation and stabilization of 53BP1 and is regulated by Src-mediated phosphorylation (20). In this study in the context of glioblastoma, our results show a negative correlation between PRMT5 inhibition and DNA repair pathways, thus reconfirming the pivotal role played by PRMT5 in DNA damage repair machinery.

TMZ, despite being the primary chemotherapeutic agent in the treatment of glioblastoma, the development of resistance to it remains a significant obstacle in achieving therapeutic efficacy. Till recently the TMZ-resistance was mainly attributed to the repair activity of O6-methylguanine-DNA methyltransferase (MGMT) (21, 32, 33). Interestingly, in our study, irrespective of the MGMT status, PRMT5 inhibition sensitized the GBMNS to TMZ. Thus, suggesting that the PRMT5 inhibition-induced sensitization of GBMNS to TMZ is MGMT-status-independent.

Owing to the extensive studies on TMZ-resistance in glioblastoma and other tumor types, researchers have identified non-MGMT related therapy resistance mechanisms such as the presence of intrinsically resistant glioma stem cell populations with an enhanced DNA repair ability (6, 23, 24), epigenetic alterations, dysregulated signaling cascades (25), thus adding additional complexity to TMZ resistance mechanism.

TMZ causes G2/M cell cycle arrest in tumor cells providing an opportunity for the cells to repair the damaged DNA and to blunt cytotoxic effect (30, 31). Here we show that the treatment of PRMT5-depleted GBMNS disrupts the G2/M cell cycle checkpoint in the TMZ-treated cells and denies the opportunity for TMZ-treated cells to repair the damaged DNA (Fig. 2). Emerging evidence underscores the critical role of HR in mediating resistance to TMZ in glioblastoma. The key HR genes are frequently overexpressed in glioblastoma cells (34). Silencing of RAD51 has been shown to enhance glioblastoma sensitivity to TMZ (35), and augment the response to radiotherapy (36). In addition to prior reports, our mechanistic study shows that PRMT5 inhibition blocks the multiple DNA damage repair mechanisms in general and HR in particular (Fig. 3). In this study TMZ treatment induced the expression of the HR gene RAD51 underling the involvement of HR in imparting TMZ resistance. With the inhibition of PRMT5, TMZ-induced RAD51 was subdued suggesting the role of PRMT5 in HR in the context of glioblastoma. This mechanistic finding linking PRMT5 to HR and HR to TMZ resistance provides a novel insight and potential solution to overcome TMZ resistance in glioblastoma because, in the combination treatment, as PRMT5 inhibition severely affects the HR repair pathway (Fig. 5), it sensitizes GBMNS to TMZ treatment.

As PRMT5 is a druggable target for glioblastoma (14), many PRMT5 inhibitors have been developed. LLY-283 is a selective SAM-competitive nucleoside inhibitor of PRMT5 and it demonstrates good brain penetration and significantly prolongs survival in mice with orthotopic glioblastoma models (15). The promising preclinical results suggest that LLY-283 could be a valuable therapeutic agent for treating glioblastoma and possibly other cancers.

Since our results show enhanced anti-tumor efficacy when LLY-283 is combined with TMZ, our study not only highlights the importance of LLY-283 for clinical use but also the potential solution to overcome TMZ resistance in cancerous conditions in general and glioblastoma in particular. Overall, this study is the first of its kind that delineates the mechanistic and clinical relevance of PRMT5 in TMZ resistance in glioblastoma.

## Data Availability

RNA-seq results have been deposited in the GEO database (https://www.ncbi.nlm.nih.gov/geo/query/acc.cgi?acc=GSE286560)

## Key Points

- TMZ-induced apoptosis is enhanced in PRMT5-inhibited GBMNS.
- PRMT5 inhibition suppresses the DNA repair ability of GBMNS.
- *In vivo,* the antitumor efficacy of TMZ is enhanced by LLY-283 treatment.
